# Self-Powered Intelligent Human-Machine Interaction for Handwriting Recognition

**DOI:** 10.34133/2021/4689869

**Published:** 2021-04-01

**Authors:** Hang Guo, Ji Wan, Haobin Wang, Hanxiang Wu, Chen Xu, Liming Miao, Mengdi Han, Haixia Zhang

**Affiliations:** ^1^National Key Laboratory of Science and Technology on Micro/Nano Fabrication, Peking University, Beijing 100871, China; ^2^Academy for Advanced Interdisciplinary Studies, Peking University, Beijing 100871, China

## Abstract

Handwritten signatures widely exist in our daily lives. The main challenge of signal recognition on handwriting is in the development of approaches to obtain information effectively. External mechanical signals can be easily detected by triboelectric nanogenerators which can provide immediate opportunities for building new types of active sensors capable of recording handwritten signals. In this work, we report an intelligent human-machine interaction interface based on a triboelectric nanogenerator. Using the horizontal-vertical symmetrical electrode array, the handwritten triboelectric signal can be recorded without external energy supply. Combined with supervised machine learning methods, it can successfully recognize handwritten English letters, Chinese characters, and Arabic numerals. The principal component analysis algorithm preprocesses the triboelectric signal data to reduce the complexity of the neural network in the machine learning process. Further, it can realize the anticounterfeiting recognition of writing habits by controlling the samples input to the neural network. The results show that the intelligent human-computer interaction interface has broad application prospects in signature security and human-computer interaction.

## 1. Introduction

The human-machine interface represents an intuitive and effective approach to bridge the communications between human and machine equipment. With the deepening of research, the solution of human-machine interaction has expanded from usual control terminals such as keyboards and touch panels to other advanced portable devices [1–8]. For example, people can directly interact with mobile phones and smart devices through voice. In addition, it is possible to control the machine by detecting people's intentions through electroencephalogram. Although many research groups have developed a variety of gesture recognition devices, such as smart gloves, some of these technologies have some limitations in practical applications, including the difficulty in identifying and detecting subtle features and the requirement on external energy supply [9–17]. Among various sensing mechanisms, triboelectric-based electronic devices surpass other resistive, capacitive, piezoelectric, and photoelectric due primarily to the feature of high output voltages and effective operation without external energy supply [18–32].

As for the signals of the human-machine interface, handwritten signature signals represent one of the most important personal characteristics in modern society. In handwriting recognition, the conventional process begins with converting the mechanical signal into an image signal, followed by scanning the image signal for digital storage or recognition. These two signal conversion processes cause the loss of a large amount of original handwritten signals, thereby leading to unreliability of handwriting recognition and authentication. The triboelectric nanogenerator converts mechanical signals into electrical signals and harvests mechanical energy by storing electrostatic charge, thus serving as ideal devices for recording handwritten signals [33–35]. Generally, self-powered active sensors based on triboelectrification require a digital array configuration (i.e., digital sensing mechanism) to detect mechanical signals [36–41]. A large number of electrodes will introduce difficulties in signal reading and data backend processing. An alternative approach (i.e., analog sensing mechanism) exploits only 2 pairs of edge electrodes, with a relatively low detection area and resolution. Recently, a research group proposed to combine digital and analog sensing mechanisms to compromise the number of electrodes, effective area, and resolution [42, 43]. Such device configurations allow the human-machine interaction system based on triboelectricity to realize high precision through a small number of electrodes.

Recently, Lee et al. demonstrated that the combination of active sensors and machine learning can carry out advanced human-machine interaction and realize high-precision augmented reality/virtual reality (AR/VR) applications [44–51]. These works inspired us to combine active sensors with machine learning for smart human-machine interaction and handwriting recognition. Here, we use a triboelectric nanogenerator with a horizontal-vertical symmetrical arrayed electrode to record the triboelectric signal of handwritten English letters, Arabic numeral, and Chinese character. The *K*-nearest-neighbour (KNN) algorithm serves as an effective neural network classification method to improve the accuracy and accuracy of classification and recognition, due to its insensitivity to outliers (random noise signals) [52–55]. Using the neural network of the KNN classifier algorithm to study the triboelectric signal enables the successful recognition of different handwritten characters. Furthermore, we used the principal component analysis (PCA) algorithm to preprocess the multidimensional electrode signal data for dimensionality reduction, thereby reducing the complexity of the process of building neural networks in machine learning [56]. The results show that the intelligent handwriting recognition system as a human-machine interaction interface has broad application prospects in personal information recognition and anticounterfeiting signatures.

## 2. Concept

In the human body's perception system, the perception and processing of information rely on receptors and neural networks distributed throughout the organism. These networks closely and effectively solve complex real-world perception problems. The receptors capture external stimuli and environmental information and then transmit them to the nervous system for intelligent learning and processing. Building artificial systems that can sense and process external stimuli similar to living organisms is very important for future intelligent robots and human-machine interfaces.

**Figure 1:**
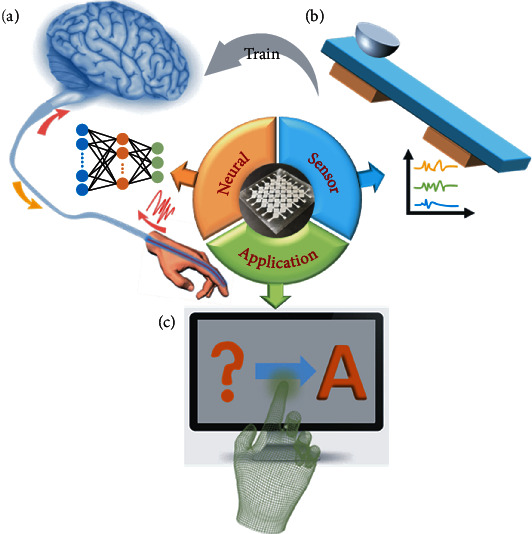
Schematic of the human-machine interface based on an intelligent handwriting recognition system: (a) artificial neural networks inspired by biological perception systems; (b) active sensor based on single-electrode triboelectric nanogenerators; (c) smart recognition application of handwritten characters.

Inspired by the sensory system of living organisms, we adopt single-electrode triboelectric nanogenerators to sense external stimuli (Figure 1(b)). When mechanical stimulation occurs on the surface of the device, the device will generate a corresponding triboelectric signal spontaneously. At the same time, the rapid development of machine learning algorithms in the field of artificial intelligence (AI) provides a brand new solution for the realization of intelligent sensing functions (Figure 1(a)). For specific sensor applications, using appropriate learning models can extract comprehensive information for sensors. Classification and recognition of handwritten characters are possible through the training of capture and output of different handwritten character signals. The handwriting recognition system proposed here can (1) act as a human-machine interaction interface, (2) yield a database about handwritten characters through more training samples, and (3) create opportunities for establishing intelligent systems that integrate perception and feedback functions.

## 3. Results and Discussions

**Figure 2:**
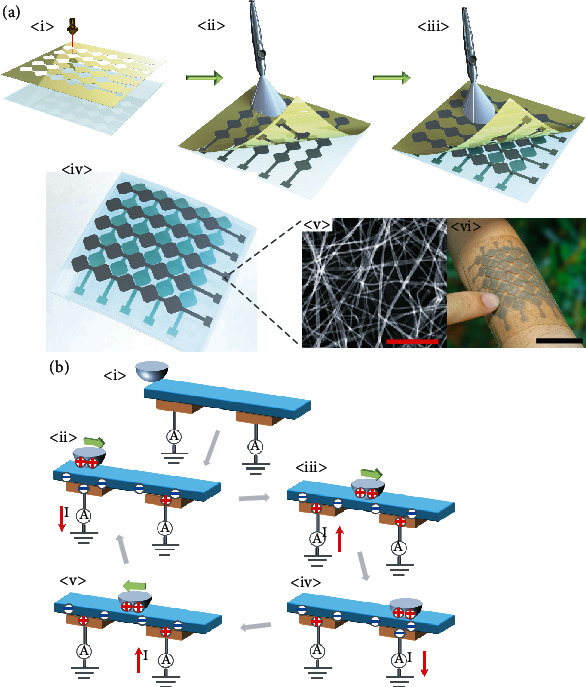
(a) Schematic diagram and preparation process for the device. <i>-<iii> Patterned PDMS-AgNW electrode spray preparation process. <iv> Schematic showing the detailed structure of the device. <v> SEM photo of AgNWs on PDMS flexible substrate (the scale bar is 1 *μ*m). <vi> Optical image of the device attached to the skin for handwritten character sensing (the scale bar is 4 cm). (b) <i>-<v> A illustrative diagram of the triboelectric signal of handwritten characters based on triboelectric nanogenerators.

Figure 2(a), <i>-<iii> shows an illustrative diagram of the preparation process of the device part of the intelligent human-machine interaction system. As demonstrated in Figure 2(a), <i>, a laser is used to cut the polyimide (PI) film into a designed electrode array. Among them, each electrode is formed by connecting five square patterns, and the gap between the electrode and the electrode exactly matches the square pattern. After attaching a PI mask onto a polydimethylsiloxane (PDMS) substrate, a spray gun sprays the silver nanowire (AgNW) solution evenly on top of the PI-PDMS structure. At this time, AgNWs will form a layer of an interlaced conductive network on the surface of PI-PDMS. Because the PI mask can be easily torn off from the PDMS substrate (Figure 2(a), <ii>), the designed patterned electrode array will be left on the PDMS substrate. Further, spin-coated PDMS on the surface of AgNWs isolates the first electrode arrays from the second electrode arrays. Afterwards, the upper electrode array is sprayed again using a PI mask, as demonstrated in Figure 2(a), <iii>.

Figure 2(a), <iv> demonstrates an illustration of the device, indicating that the pattern of the two-layer AgNW conductive network array has been specially designed. Therefore, the two electrode patterns are complementary to each other to form a crisscross structure. We designate the upper layer of AgNW conductive network electrodes as bit electrodes (electrodes B1-B5) and the lower layer of AgNW conductive network electrodes as word electrodes (electrodes W1-W5). Figure 2(a), <v> shows the SEM image of the conductive network of silver nanowires. It can be seen that the AgNWs are interlaced with one another, which also increases the overall flexibility, stretchability, and transparency of the device. The soft feature of the PDMS substrate yields a conformal interface between the device and human skin. Figure 2(a), <vi> demonstrates the physical photo of a device placed on the wrist of a human.

Figure 2(b) demonstrates the working principle regarding the intelligent human-machine interaction system. When a slider (*e.g.*, human finger) contacts with the top of the device, contact electrification occurs (as shown in Figure 2(b), <i>-<ii>). Because PDMS is strongly tribonegative (*i.e.*, attracting electron), it usually carries negative charges on the surface while countering objects bearing positive ones. As a result, a contact electrification phenomenon leads to “a signal source” for powering, eliminating the need for additional powering.

As displayed in Figure 2(b), <ii>, when a slider begins to slide across an electrode, positive charges on it will progressively balance negative charges on the surface of the PDMS substrate. This leads to current from the electrode to the ground. During the process of approximation of the slider to electrodes, the electric field is restrained between the slider and the PDMS. In the meantime, electrode outputs will be unaffected from the negatively charged PDMS upper layer; therefore, no net current flow presents in the circuit. Afterwards, the slider moves away from the electrode (Figure 2(b), < iii>); the negatively charged PDMS introduces positive charges on the corresponding electrode. As the slider finishes sliding over an electrode and is moving towards adjacent another (Figure 2(b), <iv>), an identical process will take place, as current flows from the electrode into the ground. The process shown in Figure 2(b), <iv>-<v> is a reversed process compared with that shown in Figure 2(b), <ii>-<iii>. Hence, we can determine the successive motion trajectory and status of the object on the device by measuring the sequence and number of peaks in signals on electrodes. Furthermore, we are able to obtain the speed and acceleration of the object's motion on top of the device.

**Figure 3:**
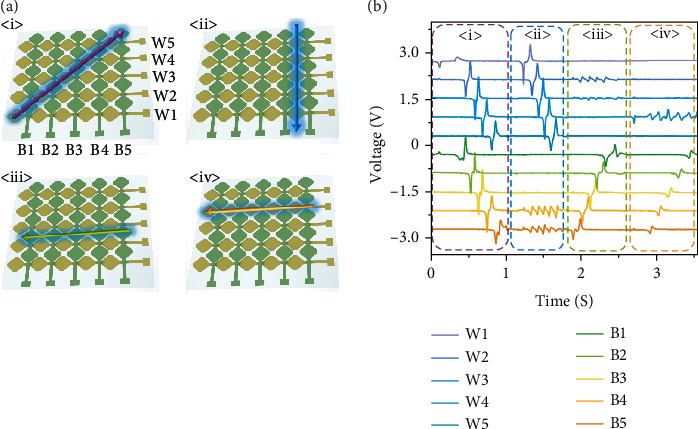
(a) Schematic diagram of different trajectories on top of device. (b) Time-domain voltage output generated by the word electrodes and bit electrodes corresponding to the 4 basic tracks in (a).

Figure 3(a) presents an illustrative diagram of the finger moving along four basic paths. As the object moves on top of the device, multiple word electrodes and bit electrodes will go across. As a result, inferring the trajectory of finger movement is possible through the number of triboelectric signal peaks on different electrodes and the sequence of appearance. Figure 3(b) corresponds to the triboelectric signals of the four different trajectories in Figure 3(a). Figure 3(a), <i> displays the motion of a finger across the 45° angle. The finger moves across ten electrodes successively (electrode B1 to electrode B5 as the bit electrodes and electrode W1 to electrode W5 as the word electrodes). As shown in the electrical output to the right, the resulting waveform matches quite well with the finger. Figure 3(a), <ii> shows the motion of the finger across the vertical direction. In the electrical outputs, there are five voltage peaks between electrode B4 and electrode B5 with respect to five patterns between electrode B4 and electrode B5. The finger continuously moved across each word electrode W1 to W5, leading to the voltage peaks on the electrode W1 to electrode W5 appearing one by one.

Figure 3(a), <iii> shows the motion of a finger along the horizontal direction. The electrical output on the right shows that 5 voltage peaks on the W2 and W3 electrodes present with respect to 5 patterns between the electrode W2 and electrode W3. The finger successively moves across through each bit electrode B5 to B1; therefore, the voltage peaks between electrode B5 and electrode B1 are generated continuously. Track <iv> demonstrates the motion of a finger along a single electrode (W4) on top of the device. Electrical output to the right shows that 5 voltage peaks exist on the W4 electrode with respect to 5 patterns on the W4 electrode. From bit electrodes B5 to B1, the finger moves across each electrode one by one, so the voltage peaks on the B5 to B1 electrodes present one by one. Although trajectory <iii> is very similar to trajectory <iv>, they are still distinguishable, thereby suggesting a reliable basis for the intelligent handwriting recognition system.

**Figure 4:**
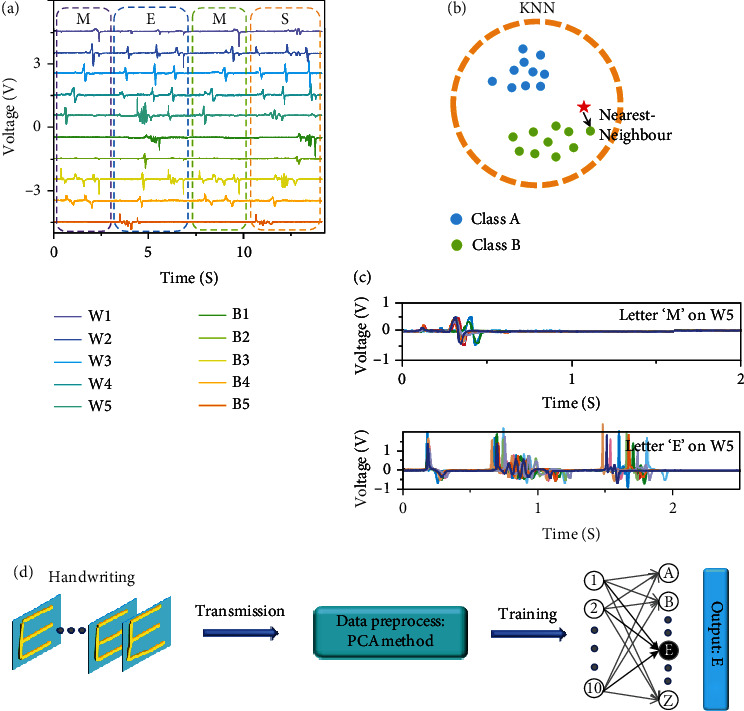
(a) Write English characters “M,” “E,” “M,” and “S” in sequence on the device. The corresponding signals generated by the word electrodes and bit electrodes. (b) Schematic diagram of KNN neural network algorithm. (c) Ten sets of handwritten signals of letter M and letter E on electrode W5. (d) The principle diagram of feature extraction and feature learning of a hardware-based intelligent handwriting recognition system.

Such trajectory data form a basis for further exploration of the triboelectric signals that correspond to complex trajectories. To this end, collecting the triboelectric signals by handwriting English letters on the surface of the device is necessary. The device consists of a 5 by 5 array of electrodes, with a total of 25 pixels. The number of pixels is sufficient to decompose 26 English letters into different trajectories.  Figure 4(a) shows the triboelectric signal diagrams on the corresponding word electrode and bit electrode, associated with English letters of “M,” “E,” “M,” and “S” written in sequence on the surface of the device. The signal diagrams prove the feasibility of decomposing complicated trajectories of English letters. In these cases, each basic trajectory can yield a unique sequence of triboelectric signals. Since all English letters are based on a collection of multiple basic trajectories, the triboelectric signal diagram corresponding to each English letter is also unique. This creates good conditions for extracting feature vectors and intelligent recognition through machine learning.

Figure 4(a) additionally shows that the triboelectric signals of the same letter “M” can have subtle differences due to the inaccurate nature of handwriting. In order to study the inaccuracy of handwritten triboelectric signals, Figure S1 shows the signal diagrams of 10 groups of different letter “M” and letter “E” traces on each electrode.  Figure 4(c) shows the signal of 10 groups of letters “M” and “E” on electrode W5. It can be seen from Figure 4(c) that although the handwritten signal corresponds to the same letter, the triboelectric signal still has slight differences (e.g., the time delay between the signal peaks) due to different writing habits. 10 sets of electrode data appear in Figure S1. At the same time, for the same letter, the key characteristics of the triboelectric signal (including the number of signal peaks and time series) are consistent. However, because the original writing trajectory is completely different between letters, the key features of the corresponding triboelectric signals are also completely inconsistent. Such features make it possible for supervised machine learning to train neural networks that can be intelligently identified.

Therefore, repeatedly writing the same letter can yield multiple groups of different triboelectric signal sequences, which serve as samples for further extraction of feature vectors to train artificial neural networks. It can quickly and accurately recognize the handwritten letters based on the trained artificial neural network.  Figure 4(b) shows the schematic diagram of the KNN neural network algorithm. As the KNN neural network algorithm performs classification and recognition by comparing the difference between the input feature vector and the feature vector in the model within a certain range, selecting an appropriate range of **k** values allows for high-precision intelligent identification and eliminates the interference of individual abnormal noise signals.

Figure 4(d) shows the intelligent recognition method of the human-computer interaction system. It is particularly worth noting that we did not directly treat the signal data obtained from the test as the output of the training neural network. The amount of data in the direct test is too large, which makes the training volume extremely complicated. Therefore, we use the PCA dimensionality reduction algorithm to decrease the dimensionality of the test data first. The main point of the PCA algorithm is to calculate the eigenvectors and eigenvalues of the variance and covariance matrix in the data. The eigenvalues are sorted, and the eigenvectors that have little impact on the structure are discarded to reduce the dimensionality of the data. In addition to greatly reducing the computer computing overhead when training the neural network, it can also mitigate the impact of noise on the results. The PCA algorithm codes we used are presented in the supplementary note.

In the neural network trained by the KNN algorithm, we can customize the neural network we need according to specific needs. As shown in Figure S2a, we can mark the handwritten triboelectric signals of different people as different training samples. Since different people have different writing habits, we can divide Class B in Figure 4(b) into Class B1 and Class B2 according to different writing habits. Figure S2b-c shows the signals on the electrodes B1-B5 when different people write the letter “M.” If the difference of these triboelectric signals is artificially distinguished, a very complicated threshold formula needs to be set. With the help of neural network algorithms, the computer can automatically calculate the characteristic differences of the input signal and perform automatic classification. Therefore, the intelligent handwriting recognition system can not only recognize the traces of written letters but also has great potential for anticounterfeiting recognition.

**Figure 5:**
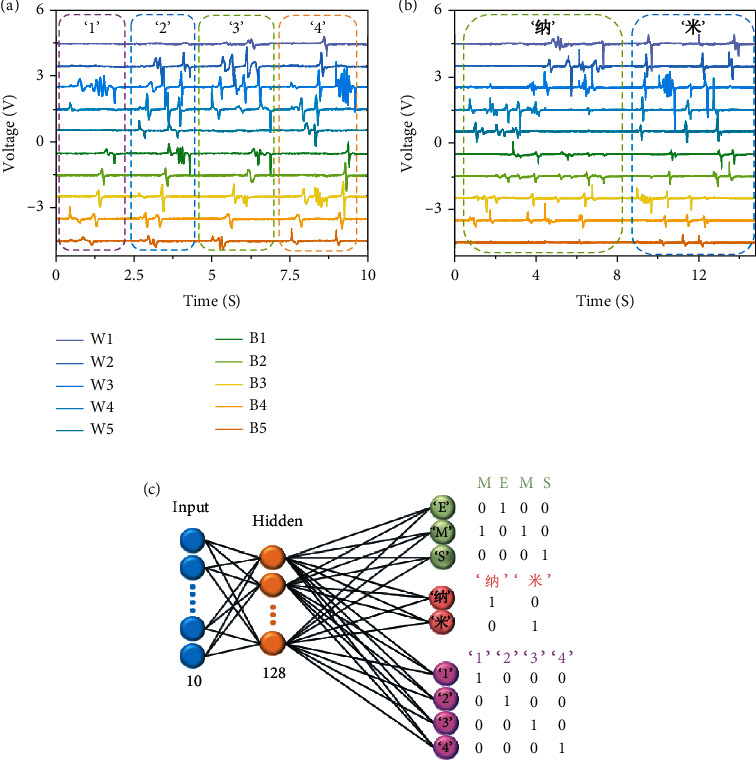
(a) When writing Arabic characters “1,” “2,” “3,” and “4,” the corresponding timing signal diagram. (b) Writing the Chinese characters “纳” and “米” in sequence, the corresponding timing signal diagram. (c) Illustration of associating the handwritten character timing signal array with a KNN for smart identification.

The machine learning algorithm can expand the scope of intelligent recognition from English letters to Arabic characters and Chinese characters.  Figure 5(a) shows the triboelectric signals on each electrode, corresponding to 4 Arabic characters “1,” “2,” “3,” and “4.” Figure 5(b) displays the triboelectric signal sequence diagram obtained by handwriting two common Chinese characters “纳” and “米” in turn. Similar to the process of recognizing English letters, multiple groups of triboelectric signal sequences through a large number of repetitions can serve as samples to train artificial neural networks. The final neural network structure, with 10 groups of neurons corresponding to 10 electrodes, appears in Figure 5(c). After debugging and optimization, we determine the optimal number of hidden neurons in the middle as 128 in order to obtain more accurate recognition performance. Finally, the number of output neurons depends on the number we need to classify. For example, setting it to 26 enables the identification of English letters; changing it to a specific number affords the capabilities in recognizing Chinese characters and Arabic characters. Combining the signal of the sensor with the artificial neural network yields an intelligent human-machine interface system, with efficient and quick communication performance between human and machine or equipment.

## 4. Conclusion

Herein, we reported a self-powered human-machine interaction interface system for smart handwriting recognition. In this system, we design a horizontal-vertical symmetrical arrayed electrode structure, with only five bit electrodes and five word electrodes to realize the identification of 25 pixels. It provides an effective method for decreasing the total electrode amount when enhancing the number of pixels. The entire device is based on a soft and transparent PDMS substrate and can integrate seamlessly to the human arm as a human-machine interaction interface. Combining the self-powered active sensing capability of the triboelectric nanogenerator with KNN's efficient classification and recognition algorithm enables the classification of handwritten characters. According to the operating principle of the TENG, the device can operate properly without external energy supply. The PCA algorithm can reduce the dimensionality of the data and reduce the computational overhead. Using the KNN neural network algorithm, the human-machine interaction interface system not only can effectively classify and identify data but also can adjust the label of the sample according to the demand to achieve anticounterfeiting identification. In general, the self-powered intelligent handwriting recognition system provides a communication window for people and equipment, machines, and virtual environments and combines triboelectric signals with machine learning methods for the next generation of intelligent systems.

## 5. Materials and Methods

### 5.1. Patterned PDMS-AgNW Thin Film Fabrication

First, the PDMS base solution (Sylgard 184, Dow Corning) is mingled with the cross-linking agent at a mass ratio of 10 : 1, respectively. Thirty minutes is then required for the mixture to be evacuated before being disposed of on a smooth glass surface to drop through the spin coating at 1000 revolutions per min which lasts 100 seconds. Afterward, the blended solution is heated at 90°C for forty-five minutes for it to cure sufficiently. Then, we further cut a polyimide (PI) mask through laser, as demonstrated in Figure 2(a), <i>, and place the modified PI mast on the surface of the PDMS. In addition, we conduct necessary oxygen plasma treatment towards the surface of the PDMS film, which was then annealed at 90°C for fifteen minutes after repeatedly spraying the AgNW ethanol solution. Ultimately, the patterned PDMS-AgNW film is obtained by peeling off the PI mask.

### 5.2. Device Fabrication

Specifically, with the two patterned PDMS-AgNWs being successfully fabricated, we first stack them vertically while also ensuring them connected by liquid PDMS. Ten minutes is required to heat the stacked thin films on a hot plate at 90°C. Lastly, in order to better protect the AgNW from being exposed, the sensor is encapsulated with liquid PDMS then cured, contributing to a total thickness of the electronic skin of 550 *μ*m.

## Data Availability

The (xls) data used to support the findings of this study are included within the supplementary materials (shared data).

## Supplementary Materials

Supplementary 1 Figure S1: voltage outputs of 10 sets of data for each electrode about letter M and letter E. Figure S2: schematic diagram of KNN algorithm for classifying different samples according to different writing habits. Supplementary Notes: PCA dimensionality reduction algorithm.

Supplementary 2 Shared data 1: Figure 3(b), i. Shared data 2: Figure 3(b), ii. Shared data 3: Figure 3(b), iii. Shared data 4: Figure 3(b), iv. Shared data 4-8: Figure 4(a). Shared data 9-12: Figure 5(a). Shared data 13-14: Figure 5(b).
