# Supplementary material for: Self-Powered Intelligent Human-Machine Interaction for Handwriting Recognition
Source: Research (Wash D C). 2021 Apr 1;2021:4689869. doi: 10.34133/2021/4689869 (PMC8035911; doi:10.34133/2021/4689869)
Supplement: Supplementary 1 — Figure S1: voltage outputs of 10 sets of data for each electrode about letter M and letter E. Figure S2: schematic diagram of KNN algorithm for classifying different samples according to different writing habits. Supplementary Notes: PCA dimensionality reduction algorithm. [file 4689869.f1.docx]

Supporting Information

**Self-Powered Intelligent Human-Machine Interaction for Handwriting Recognition**

*Hang Guo,^1^ Ji Wan,^2^ Haobin Wang,^2^ Hanxiang Wu,^2^ Chen Xu,^1^ Liming Miao,^2^ Mengdi Han,^2*^ and Haixia Zhang^1,2^**

^1^ Academy for Advanced Interdisciplinary Studies, Peking University, Beijing 100871, China.

^2^ National Key Laboratory of Science and Technology on Micro/Nano Fabrication, Peking University, Beijing 100871, China.

*Co-corresponding-authors: hanmd1990@gmail.com, zhang-alice@pku.edu.cn

Keywords: Human-Machine Interaction, Handwriting Recognition, Triboelectric Signal, Machine Learning

**1. Supplementary Figure**


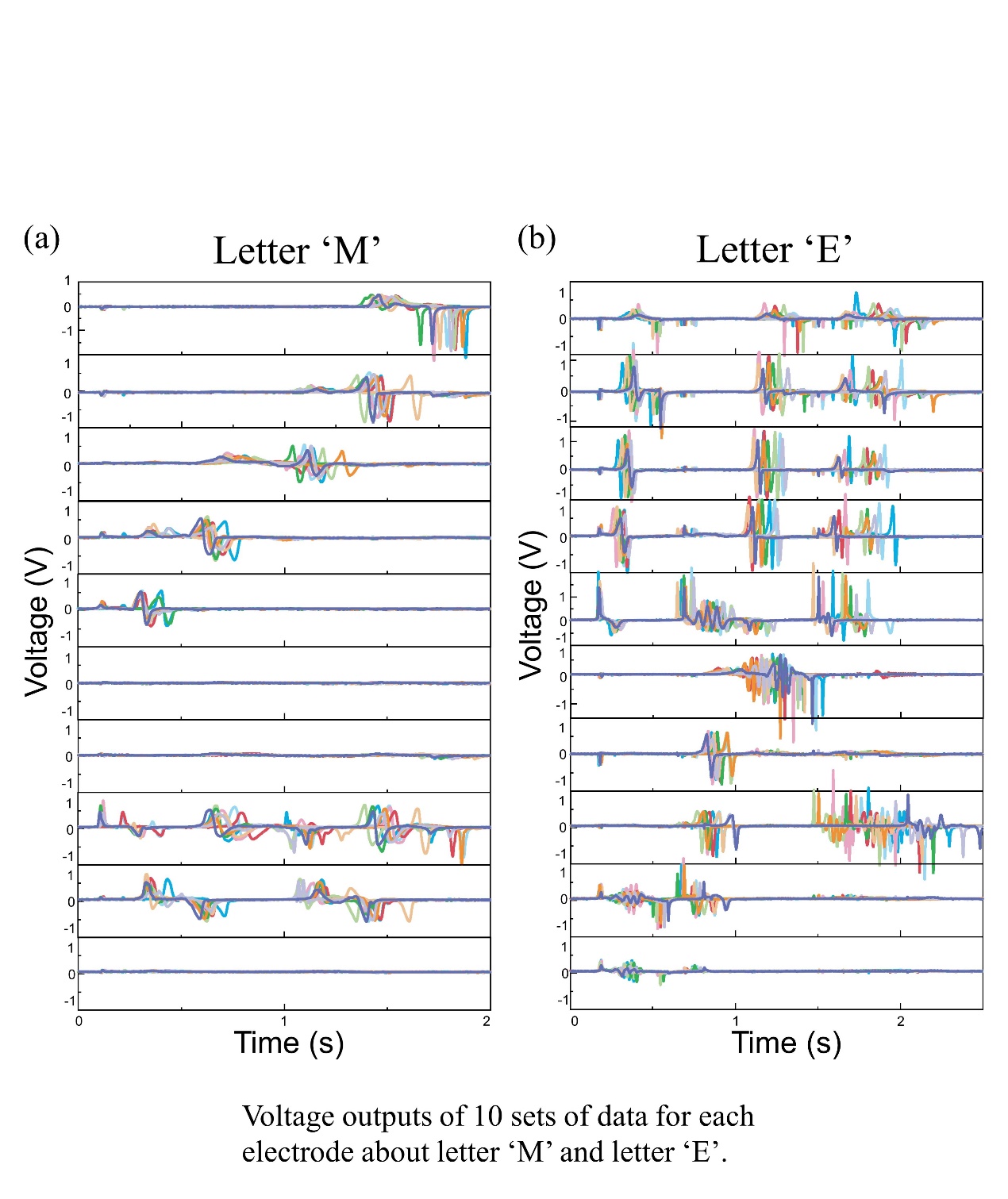


**Figure S1.** Voltage outputs of 10 sets of data for each electrode about letter ‘M’ and letter ‘E’.


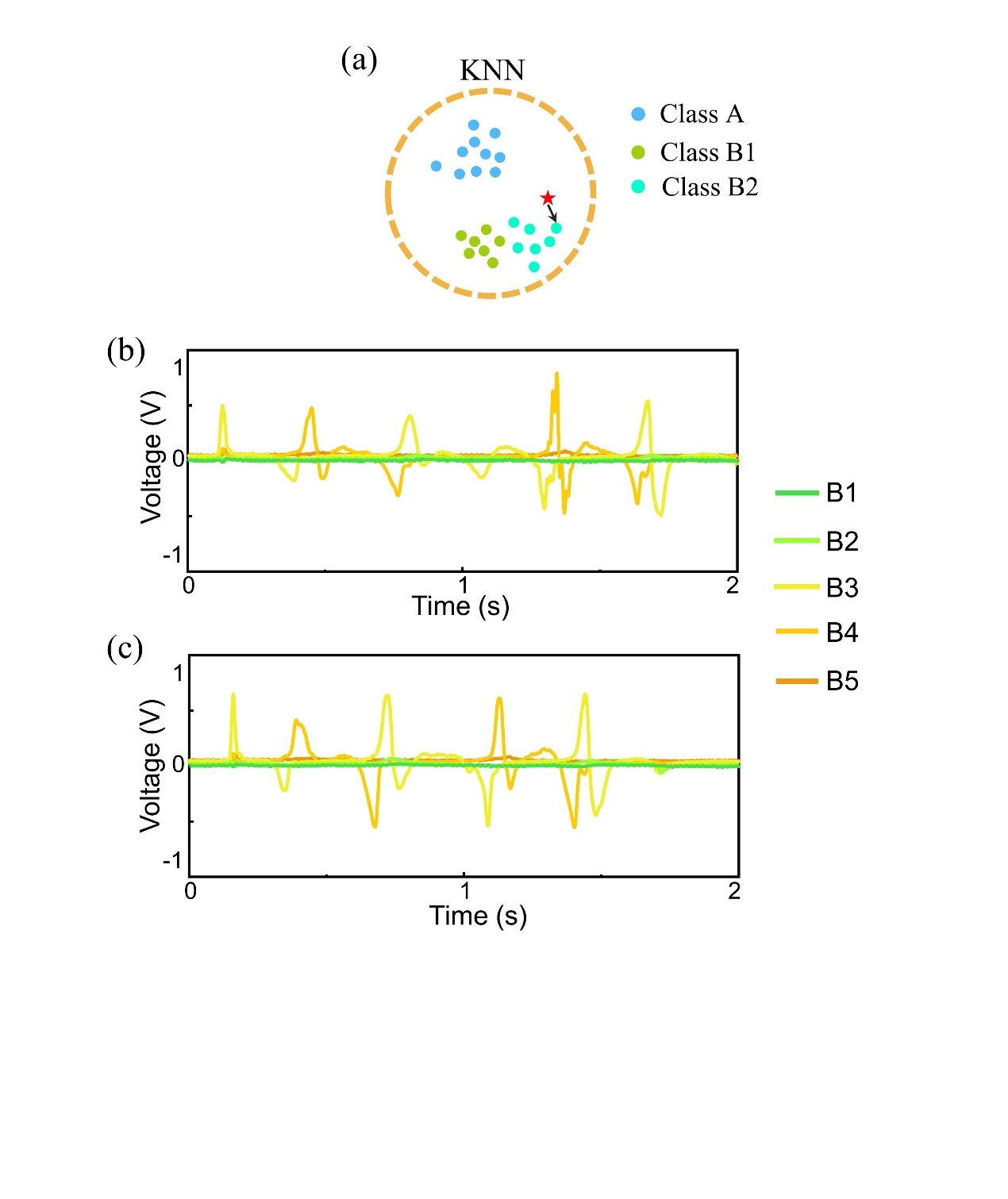


**Figure S2.** (a) Schematic diagram of KNN algorithm for classifying different samples according to different writing habits. (b)-(c) Signal diagram of letter M on electrodes B1-B5 using different writing habits.

**2. Supplementary Notes**

1. PCA dimensionality reduction algorithm

from numpy import

# Import the numpy library

# Parse text data function

#@filename file name .csv

#@delim A spaced apart manner between each row of data of different characteristics. The default is the tab key '\t'

def loadDatset (filename,delim='\t')

fr=open (filename)

# Open csv file

stringAr= [line.strip().split(delim) for line in fr.readlines()]

# Separate the characteristics of each line in the text and store them in the list

datAr= [map (float.line) for line in stringAr]

# Use the map() function to map the data value of each row in the list to float type

return mat(datAr)

# Convert the list of float data values into a matrix and return

# PCA feature dimension compression function

#@dataMat Data set matrix

#@topNfeat Feature dimensions (N) that need to be retained

def pca(dataMat, topNfeat=N):

meanVal=mean(dataMat, axis=0)

# Find the mean of each column of the data matrix

meanRemoved=dataMat-meanVals

covMat=cov(meanRemoved, rowvar=0)

eigVals, eigVects=linalg.eig(mat(conMat))

# Calculate the eigenvalues and corresponding eigenvectors of the covariance matrix

eigValInd=argsort(eigVals)

eigValInd=eigValInd[:-(topNfeat+1):-1]

redEigVects=eigVects[:,eigValInd]

# Extract the eigenvectors corresponding to the index of the N eigenvalues with the largest eigenvalues to form a compression matrix

lowDDataMat=meanRemoved*redEigVects

# Multiply the data matrix after removing the mean by the compressed matrix, and convert it to a new space, reducing the dimension to N

reconMat=(lowDDataMat*redEigVects.T)+meanVals

# Deconstruct the original data matrix using the reduced dimension matrix

return lowDDataMat,reconMat

# Return the compressed data matrix and the matrix to deconstruct the origin
